# Magnetic Control of Fe_3_O_4_ Nanomaterial for Fat Ablation in Microchannel

**DOI:** 10.3390/ma8115429

**Published:** 2015-11-19

**Authors:** Ming Chang, Ming-Yi Chang, Wei-Siou Lin, Jacque Lynn Gabayno

**Affiliations:** 1 College of Mechanical Engineering and Automation, Huaqiao University, Xiamen 361021, Fujian, China; 2 Department of Mechanical Engineering, Chung Yuan Christian University, Chungli, Taoyuan 32023, Taiwan; andy03022@gmail.com (M.-Y.C.); hugh0322452@gmail.com (W.-S.L.); 3 Mapua Institute of Technology, Intramuros, Manila 1002, Philippines; jlfgabayno@mapua.edu.ph

**Keywords:** Fe_3_O_4_ nanoparticles, magnetic control, oleic-acid, fat ablation

## Abstract

In this study, surface modification of iron (II, III) oxide Fe_3_O_4_ nanoparticles by oleic acid (OA) coating is investigated for the microablation of fat in a microchannel. The nanoparticles are synthesized by the co-precipitation method and then dispersed in organic solvent prior to mixing with the OA. The magnetization, agglomeration, and particle size distribution properties of the OA-coated Fe_3_O_4_ nanoparticles are characterized. The surface modification of the Fe_3_O_4_ nanoparticles reveals that upon injection into a microchannel, the lipophilicity of the OA coating influences the movement of the nanoparticles across an oil-phase barrier. The motion of the nanoparticles is controlled using an AC magnetic field to induce magnetic torque and a static gradient field to control linear translation. The fat microablation process in a microchannel is demonstrated using an oscillating driving field of less than 1200 Am^−1^.

## 1. Introduction

The use of magnetic nanoparticles (NPs) for biomedical applications such as microsurgery and drug delivery is a topic that draws significant interest [1,2]. One of the challenges to magnetically controlled NPs includes finding a biocompatible material that has a large magnetic moment so it can react to an external magnetic field source. Iron oxides (IO) are one of such materials which exhibit stable magnetic properties and favorable biocompatibility. Although many practical applications of IOs can be limited by their weak surface functionality, recent investigations sought methods that can improve their surface properties for optimized movement in aqueous environments [3]. The development of surface coating to improve functionality and magnetic stability is emphasized. Surface coating IO-NPs with organic molecules or surfactants, biomolecules, and polymers or grafting with inorganic layers such as silica, metal oxide, *etc.* [4,5,6], is actively investigated. Surfactants are shown as important stabilizing agents for controlling particle size and agglomeration of NPs in a suspension.

Bare magnetic Fe_3_O_4_ NPs in suspension can be controlled to move in a low gradient and oscillating magnetic fields [7]. A velocity field created by the movement of the NPs was necessary to facilitate the microablation of a thrombus in a microchannel. Due to the oleophobic property of IO materials, the use of a similar magnetic control system to steer NPs through a fat occlusion can be difficult without surface modification. In this study, oil-soluble oleic acid (OA) is used as a coating for the Fe_3_O_4_ NPs in order to decrease their agglomeration as well as enhance lipophilicity and magnetic stability. The NPs are dispersed in long-chain OA that acts as a dense protective layer around the NPs, thereby providing a monodispersed and highly uniform particle distribution. The size distribution of surface-coated NPs was compared to bare (or uncoated) NPs using scanning electron microscope (SEM) and transmission electron microscopy (TEM) measurements. The saturation magnetization was evaluated by superconducting quantum interference device (SQUID) magnetometer and the oleic acid coating was confirmed by Fourier transform infrared (FTIR) spectroscopy. 

By injecting the OA-coated Fe_3_O_4_ NPs in a microchannel, experimental investigations were carried out to demonstrate a feasible application of the surface-modified NPs. The movement of the NPs was controlled using an external oscillating magnetic field to remove a fat occlusion in the microchannel. By demonstrating that the NPs can be transported across the immiscible barrier of the suspension medium and fat layer, the system can be utilized for targeted delivery of NPs between the bloodstream and fatty tissue membranes. 

## 2. Synthesis of Surface-Coated Nanomaterials

### 2.1. Preparation of OA-Coated Fe_3_O_4_ NPs

Bare Fe_3_O_4_ NPs were first prepared at room temperature via the co-precipitation reaction of iron (II) chloride tetrahydrate and iron (III) chloride hexahydrate with sodium hydroxide. The reaction is shown below:

FeCl_2_ + 2 FeCl_3_ + 8 NaOH = Fe_3_O_4_ + 8 NaCl + 4 H_2_O (1)

**Figure 1:**
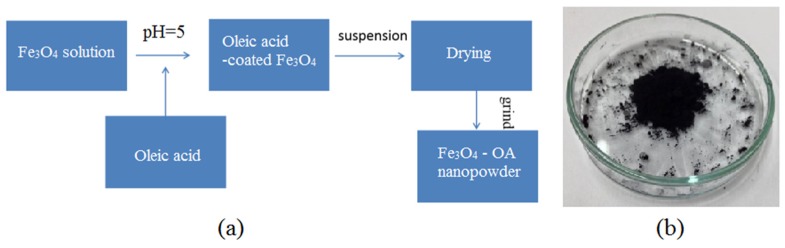
(**a**) Preparation of Fe_3_O_4_ coated with oleic acid; (**b**) Bare Fe_3_O_4_ nanopowder prepared by co-precipitation method.

Afterwards, two grams of the bare Fe_3_O_4_ nanopowders were mixed with 50 mL HCl at a pH = 5 to make a solution as illustrated in Figure 1. The solution was ultrasonicated for about 5 min before adding ethanol (20 mL) and OA (5 g) and then re-sonicated for another 30 min. The mixture was centrifuged for 30 min at 6000 rpm and oven-dried for 24 h before grinding to produce Fe_3_O_4_-OA nanopowder.

### 2.2. Characterization of OA-Coated Fe_3_O_4_ NPs

**Figure 2:**
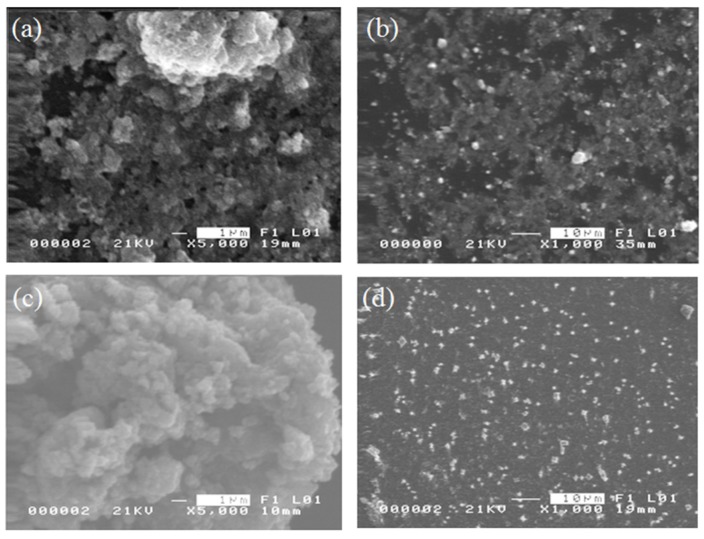
Size distribution of (**a**), (**b**) bare Fe_3_O_4_ NPs and (**c**), (**d**) Fe_3_O_4_-OA NPs investigated under scanning electron microscope (SEM).

Figure 2 shows the particle distribution of bare Fe_3_O_4_ NPs and OA-coated NPs. Due to their smaller particle size and larger surface energy, agglomeration in bare NPs is more evident. The surface-coated Fe_3_O_4_ NPs are observed to be monodispersed. The size distribution after coating is also more uniform, which can be attributed to the adsorption of the carboxyl OA on the hydroxyl Fe_3_O_4_ surface to lessen the formation of aggregates among the NPs.

**Figure 3:**
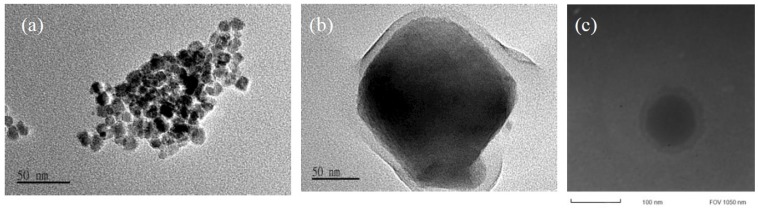
Transmission electron microscopy (TEM) images of (**a**) bare Fe_3_O_4_ NPs and (**b**), (**c**) Fe_3_O_4_-OA NPs.

Figure 3 compares the size distribution of the NPs based on TEM images. The bare Fe_3_O_4_ NPs are smaller in size, averaging from 10 to 15 nm, and form clusters as shown in Figure 3a. The Fe_3_O_4_-OA NPs are dispersed and have an average diameter of 100–150 nm as shown in Figure 3b,c. 

**Figure 4:**
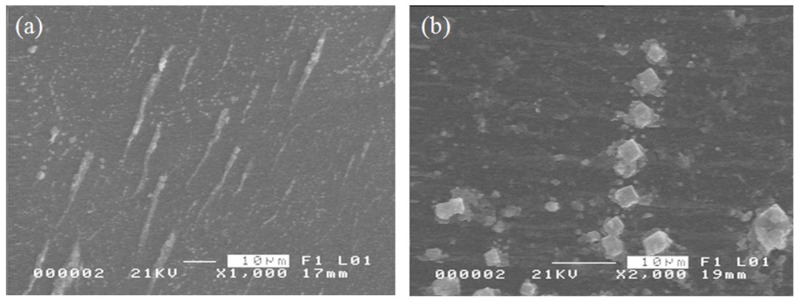
SEM images of magnetized (**a**) bare Fe_3_O_4_ and (**b**) Fe_3_O_4_-OA NPs.

To observe the response of the NPs to a static magnetic field, bare and surface-coated NPs in suspension were initially exposed to a magnetic field for about 30 s and then oven-dried. The baking temperature was set to 80 °C for 10 min. Figure 4 shows the SEM images of the bare and OA-coated Fe_3_O_4_ NPs magnetized at ~250 A·m^−1^. The bare NPs form rod-like structures 20 to 25 μm in length while Fe_3_O_4_-OA NPs form chains with an average length of 30 μm. The chain-like structures in magnetized NPs can be attributed to their magnetorheological properties, owing to the induced magnetic dipole interactions among the dispersed nanoparticles during magnetization [8,9]. As shown in Figure 2, magnetic NPs become randomly dispersed in the absence of the magnetic field. 

**Figure 5:**
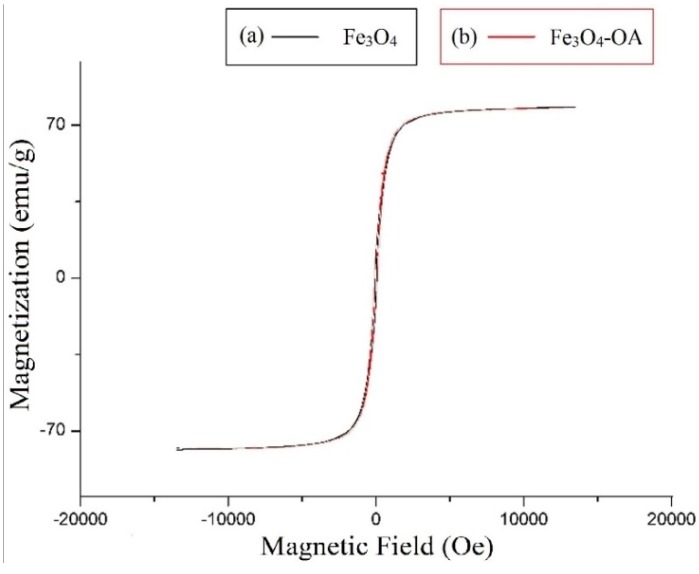
Magnetic saturation of (**a**) bare Fe_3_O_4_ and (**b**) Fe_3_O_4_-OA NPs.

Figure 5 shows the magnetization of the NPs measured using SQUID. The saturation magnetization of Fe_3_O_4_-OA is constant at 70 emu/g while the coercivity in both bare and coated samples is negligible, which reveals that the magnetic property of the Fe_3_O_4_ NPs was not altered by the surface coating.

**Figure 6:**
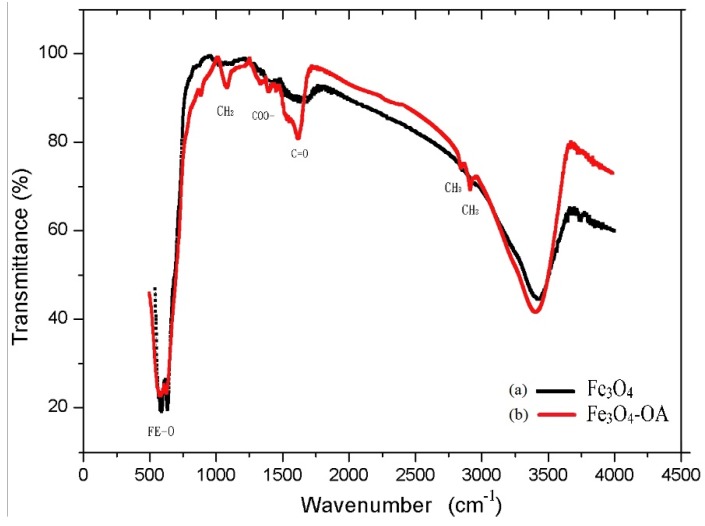
FTIR images of (**a**) bare Fe_3_O_4_ and (**b**) Fe_3_O_4_-OA NPs.

The FTIR results in Figure 6 show that at 2925 cm^−1^ and 2853 cm^−1^, there is –CH_2_ and –CH_3_ absorption, respectively, from the OA molecule. The OA double-bond and –COO absorption are also confirmed at 1623 and 1408 cm^−1^.

## 3. Magnetically Controlled Motion of Nanoparticles

Assuming that the electrostatic and van der Waals forces are negligible, a generalized formulation based on the magnetic field gradient can be used to describe the movement of the NPs in a viscous fluid. The linear translation of the NPs along the microchannel responds to a magnetic force (*F_x_*) given by [7]:

(2) $$\vec{F_{x}}={\text{μ}}_{0}\text{χ}H_{y}V\cdot \frac{\partial {\vec{H}}_{y}}{\partial x}$$

where μ_0_ is the vacuum permeability, χ is the material susceptibility, *V* is the volume of the surface-coated NPs, *H_y_* is the magnetic field strength, and $\frac{\partial {\vec{H}}_{y}}{\partial x}$ is the gradient field.

Therefore, the linear velocity of spherical NPs along the direction of the gradient subjected to a hydrodynamic Stokes’ drag force, ${\vec{F}}_{d}=-3\text{π}d\text{η}\vec{v}$, can be expressed as:

(3) $$\vec{v}=\frac{{\text{μ}}_{0}\text{χ}V}{3\text{π}d\text{η}}H_{y}\cdot \frac{\partial {\vec{H}}_{y}}{\partial x}$$

where *d* is the equivalent diameter of a sphere with the same volume as the NPs and η is the liquid viscosity.

The expression shows that the terminal velocity of the NPs changes linearly with the quantity $\left(H\frac{\partial H}{\partial x}\right)$, which is a coupled effect of the driving magnetic field and the gradient field.

The rotation of the NPs is dependent on the interaction of the induced magnetization to the oscillating magnetic field. The magnetic torque can be expressed as [7]:

(4) $${\text{τ}}_{m}=V\frac{{\text{χ}}^{2}}{2\left(2+\text{χ}\right)}{\text{μ}}_{0}H^{2}\sin\left(2\text{θ}\right)$$

where θ is the angle between the magnetic dipole moment of the NPs *m* and *H*. The expression shows that the magnetic torque quadratically changes with *H.* For a NP of radius *r*, the rotation is subjected to a viscous drag force, ${\vec{\text{τ}}}_{D}=3{\text{π}}^{2}\text{η}r^{4}\vec{\text{ω}}$, which counterbalances the magnetic torque. Therefore, the rotation speed of the NPs can be expressed as:

(5) $$\text{ω}=\frac{Vx^{2}{\text{μ}}_{0}H^{2}\sin\left(2\text{θ}\right)}{3{\text{π}}^{2}\text{η}r^{4}}$$

**Figure 7:**
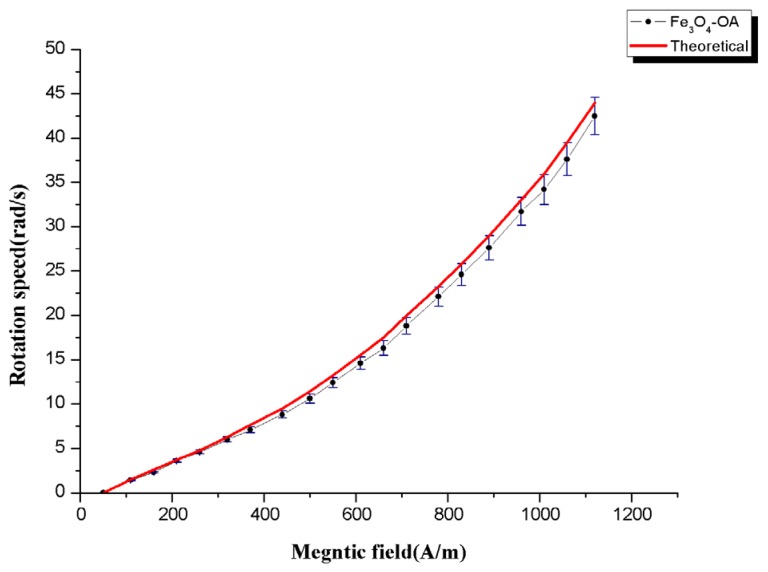
Rotation speed of the Fe_3_O_4_-OA as a function of the oscillating magnetic field.

Equation (5) implies that the NPs can be rotated in a controllable fashion by modulating the time-dependent magnetic field source. Thus, the movement of the NPs can be stopped or resumed at will by simply switching the alternating current source. A common optical inspection camera (X-Stream XS-3, Intergrated Design Tools, Tallahassee, FL, USA) attached to an optical microscope was used to track the movement of the surface-coated NPs under the action of a specific magnetic field. Video recordings of the moving nanostructures were captured at 100× magnification and subsequently loaded onto a central computer. Figure 7 shows the nonlinear dependence of the rotation speed to *H*, which was also verified experimentally on Fe_3_O_4_-OA NPs. 

## 4. Application of Fe_3_O_4_-OA for Fat Microablation

The application of the OA-coated NPs is investigated for fat removal. The Fe_3_O_4_-OA suspension (0.03 wt %) was injected into a microchannel (width = 0.8 mm, length = 25 mm) as shown in Figure 7. Because of the gradient field, the magnetic NPs are steered towards the target consisting of a fat occlusion on the left-hand side of the microchannel, as shown in Figure 7a. Without a lipophilic surface activator such as the oleic acid to which the NPs are attached, magnetic guidance of the NPs through the immiscible boundary of the suspension and fat occlusion is expectedly difficult. Surface modification of the Fe_3_O_4_ NPs by their attachment to the OA chains weakens the surface tension along the boundary, thereby allowing the transport of NPs across the microchannel. 

**Figure 8:**
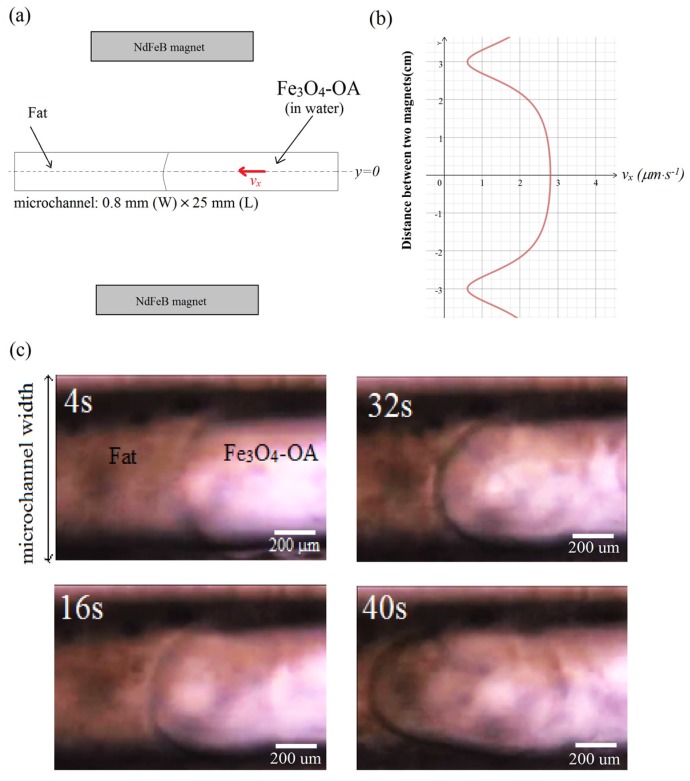
(**a**) External magnetic field source from two permanent magnets. The oscillating field source is not shown; (**b**) Calculated velocity distribution of magnetic Fe_3_O_4_-OA NPs due to the static magnetic field; (**c**) Velocity streamlines between the suspension and fat layer along the microchannel.

A magnetic control system consisting of an oscillating field source (~1200 A·m^−1^) and a static magnetic field (80 A·m^−1^) was used in the investigation. A magnetic coil connected to an AC source was used to generate the oscillating field. The coil was positioned under the microchannel stage. The static magnetic field was produced by two NdFeB permanent magnets that were positioned as shown in Figure 8a, and were separated by a distance of 6 cm. Using this configuration, the velocity distribution of the NPs is shown in Figure 8b, revealing a maximum speed in the central vicinity of the microchannel, which then tapers off, almost approaching the zero value near the location of the two magnets. Because the microchannel width is narrower compared to the gap that separates the two magnets, the NPs can then be assumed to have uniform velocity as they move in the magnetic field region. The optical inspection images in Figure 8c reveal the formation of a parabolic velocity streamline as the NPs move along the microchannel. The streamline delineates the boundary separating the dense fat layer on the left-hand side of the microchannel and the suspension carrying the OA-coated NPs injected on the right-hand side of the microchannel. Such streamlines were absent upon injection of bare NPs, indicating immobility and transport difficulty of the NPs through the fat layer in the absence of the OA coating. 

Because the size of the surface-coated Fe_3_O_4_ particles is very small, roughly at 100–150 nm, the bolus caused by the ablation process in Figure 8c is expected to be in the same range of several hundred nanometers and is, therefore, difficult to observe directly. In comparison, the diameter of human capillaries is roughly several (six or seven) micrometers; thus, the bolus released into the bloodstream by this process can pose very minimal risk for a heart attack or an induced stroke.

Moreover, the flow behavior of the magnetic field-driven Fe_3_O_4_-NPs along the fat layer was observed to be laminar. Between the time that the NPs were injected until *t* = 4 s, the viscosity effect from the wall boundaries was negligible as revealed by the small radius of the meniscus layer separating the suspension and the fat target. As the NPs move towards the target from *t* =16 s to *t* = 40 s, shear stresses from the microchannel walls become significant, hence the evident parabolic streamline between the two layers. Such a streamline would suggest that there is a dense concentration of the OA-coated NPs where the velocity is a maximum, which is near the center of the microchannel. Thus, the increased density and speed of the coated NPs can enhance surface interaction as they rotate to remove the fat target. The calculated flow rate speed in the middle is nearly constant at *v_x_* ≈ 3 μm·s^−1^. This is equivalent to a Reynolds number of less than 0.001, suggesting a laminar flow.

## 5. Conclusions 

Oleic acid-coated Fe_3_O_4_ NPs were prepared in suspension to surface-functionalize the NPs. The magnetic properties of OA-coated Fe_3_O_4_ nanoparticles were characterized, revealing that their saturation magnetization was preserved and the particle aggregation was minimized because of the OA coating. Surface-modified Fe_3_O_4_ NPs that were injected in an occluded microchannel were able to demonstrate controlled targeting through a fat layer. The removal of fat occlusion using an oscillating magnetic field to control the NPs was demonstrated. This work can be used as a model system for targeted delivery of drug-loaded magnetic NPs from the bloodstream to fatty tissue membranes.
